# Bioaccumulation and Human Health Risk Assessment of Potentially Toxic Elements in Commercial Fish Species (*Oreochromis niloticus, Clarias gariepinus, Mugil cephalus*) from Slaughterhouse Wastewater-Impacted Rivers in Nigeria

**DOI:** 10.3390/ijerph23070827

**Published:** 2026-06-23

**Authors:** Onyedikachi Uchechi Bliss, Edene Osemudiamen Anao, Paul Promise Chibuike, Ugorji Chizoba Agatha, Peter Chinedu Agu, Emmanuel Anuoluwapo Oke

**Affiliations:** 1 Department of Biochemistry, Michael Okpara University of Agriculture, Umudike 440101, Nigeria; chibuike.paul@mouau.edu.ng (P.P.C.); ugorjichizoba71@gmail.com (U.C.A.); 2 Sustainable Materials Research Centre, University of Mohammed IV Polytechnic, Ben Guerir 43150, Morocco; 3 Department of Environmental Management and Toxicology, Faculty of Life Sciences, University of Benin, Benin City 300213, Nigeria; osemudiamen.anao@uniben.edu; 4 Department of Biochemistry, College of Science, Evangel University, Akaeze 481116, Nigeria; pc.agu@evangeluniversity.edu.ng; 5 College of Pharmaceutical Sciences, Southwest University, Chongqing 400715, China; 6 School of Chemical and Metallurgical Engineering, University of the Witwatersrand, Johannesburg 2050, South Africa; emmanuel.oke@wits.ac.za

**Keywords:** potentially toxic elements, bioaccumulation, slaughterhouse wastewater, health risk assessment, Flathead grey mullet, Niger Delta

## Abstract

**Highlights:**

**Public health relevance—How does this work relate to a public health issue?**

- Unregulated/untreated slaughterhouse wastewater introduces potentially toxic elements (lead (Pb), cadmium (Cd), and chromium (Cr)) into rivers, inhabiting fish that serve as primary protein sources for subsistence fishing communities in low- and middle-income countries.
- Chronic dietary intake of contaminated fish exposed to Pb and Cd is linked to oxidative stress-induced DNA and neurodevelopmental impairment in children, renal dysfunction, and increased carcinogenic risk.

**Public health significance—Why is this work of significance to public health?**

- This study provides a comprehensive health risk assessment from West Africa quantifying six potentially toxic elements (zinc, lead, iron, magnesium, chromium, and cadmium (Zn, Pb, Mg, Cr and Cd)) in three commercially important fish species, revealing that Pb and Cd levels in *Mugil cephalus* exceeded the EU safety limit (0.3 and 0.05, respectively) by 500%, and CD levels exceed the limit by 800%.
- Mg was employed as a geogenic tracer, reflecting the natural geological background in principal component analysis. This distinction confirmed that Pb and Cd are likely from anthropogenic sources (slaughterhouse effluent and tyre combustion), whereas Mg appears to be primary from natural sources, thereby strengthening source apportionment.

**Public health implications—What are the key implications or messages for practitioners, policy makers and/or researchers in public health?**

- Policymakers should mandate wastewater treatment at slaughterhouse facilities and enforce existing environmental regulations to prevent untreated effluent discharge into receiving water bodies.
- Public health practitioners should issue species-specific fish consumption advisories for high-risk areas, with particular emphasis on detritivorous species such as mullet (*Mugil cephalus*), which accumulate the highest lead and cadmium burdens.
- Researchers may consider using essential elements such as magnesium in multivariate analysis as potential geogenic tracers. In this study, Mg was loaded independently from anthropogenic pollutants (Pb, Cd), suggesting it could help distinguish natural background from contamination in similar settings. However, direct validation with water, sediment, or isotopic analysis is recommended.

**Abstract:**

Slaughterhouse wastewater introduces potentially toxic elements into aquatic ecosystems, yet bioaccumulation patterns in commercial fish species and associated human health risks remain underexplored in West Africa. This study quantified zinc (Zn), lead (Pb), iron (Fe), magnesium (Mg), chromium (Cr), and cadmium (Cd) in three ecologically distinct fish species—*Oreochromis niloticus* (Nile tilapia), *Clarias gariepinus* (African sharptooth catfish), and *Mugil cephalus* (Flathead grey mullet)—from two slaughterhouse-impacted rivers (Transamadi and Mgbuosimini) and a control site (Iwofe) in Rivers State, Nigeria. Metal concentrations were measured using atomic absorption spectrophotometry. Two-way ANOVA assessed species and location effects. Principal component analysis (PCA) was performed, with Mg used as a potential geogenic tracer, as its loading pattern was independent of Pb and Cd and consistent with the natural background. A Water Quality Index (WQI) classified Mgboshimini and Iwofe as having poor water quality (WQI > 75), while Transamadi had medium quality. Health risks were evaluated using estimated daily intake (EDI), target hazard quotients (THQ), and hazard indices (HI) following USEPA guidelines. Metal levels varied significantly by species and location (*p* < 0.001). Flathead grey mullet from Mgbuosimini had the highest Pb (1.50 ± 0.05 mg/kg) and Cd (0.41 ± 0.02 mg/kg), exceeding EU maximum levels for fish muscle (Pb 0.30 mg/kg, Cd 0.05 mg/kg) by 500% and 800%, respectively. PCA explained 77.5% of the variance, with Pb and Cd clustering as anthropogenic sources, while Mg loaded independently. THQ for Pb approached unity in Flathead grey mullet (0.88), and THQ for Cd reached 0.97. HI exceeded 1.0 in all species from Mgbuosimini, peaking at 2.07 in Flathead grey mullet. Uncertainty analysis (using ±SD) gave a HI range of 1.89–2.25 for this species, all above the safety threshold. Carcinogenic risk for Flathead grey mullet (3.97 × 10^−4^) approached the upper acceptable limit. Slaughterhouse effluent appears to elevate Pb and Cd burdens in fish, with detritivorous Flathead grey mullet posing the highest health risk. Exceedance of safety thresholds and HI > 1.0 indicate potential non-carcinogenic and carcinogenic risks. We recommend improved wastewater treatment and species-specific consumption advisories.

## 1. Introduction

The rapid expansion of animal protein production to meet the dietary needs of growing populations has intensified environmental concerns surrounding slaughterhouse operations worldwide [1,2]. These facilities generate vast quantities of untreated or inadequately treated wastewater containing blood, fats, proteins, pathogens, and a complex mixture of inorganic contaminants, including potentially toxic elements [3]. In developing regions where regulatory enforcement remains challenging, such wastewater is frequently discharged directly into adjacent water bodies, creating chronic pollution hotspots with implications for aquatic ecosystem health and human food safety [4,5].

Lead (Pb), cadmium (Cd), chromium (Cr), and Zinc (Zn) are of particular toxicological significance due to their environmental persistence, capacity for bioaccumulation in aquatic organisms, and potential to induce both acute and chronic health effects in exposed human populations [6,7]. Unlike organic contaminants, metals cannot be degraded by biological or chemical processes, leading to long-term accumulation in sediments and aquatic biota [8]. Chronic dietary exposure to metals has been associated with nephrotoxicity, neurodevelopmental impairment, cardiovascular disease, and carcinogenesis, with children and pregnant women representing particularly vulnerable subpopulations [9,10].

International regulatory frameworks establish permissible limits for potentially toxic elements in food intended for human consumption. The Food and Agriculture Organisation and World Health Organisation [11] set maximum permissible concentrations of 0.5 mg/kg for Pb, 0.5 mg/kg for Cd, and 40 mg/kg for Zn in fish. More recently, the European Union [12] adopted stricter standards under Commission Regulation (EC) No 1881/2006, setting maximum levels of 0.30 mg/kg for Pb and 0.05 mg/kg for Cd in fish muscle. Despite well-characterised toxicity pathways and established regulatory standards, the specific contribution of slaughterhouse wastewater to metal contamination in receiving aquatic ecosystems remains poorly quantified. A systematic review by Agoro et al. [1] identified only 12 studies examining potentially toxic elements in African water bodies receiving abattoir effluent, with none conducting a comprehensive human health risk assessment for fish consumers against international standards [13].

Magnesium as a potential geogenic tracer. Magnesium (Mg) is an essential element whose concentrations in aquatic systems are primarily controlled by natural geological weathering rather than anthropogenic inputs [14]. Recent studies have successfully applied integrated receptor modelling (e.g., positive matrix factorisation) together with thermodynamic and isotopic tools to quantitatively distinguish geogenic from anthropogenic controls on trace-element mobility in contaminated water and sediment systems [15,16]. When analysed concurrently with potentially toxic elements, Mg may serve as a natural geochemical tracer that helps discriminate between pollution-derived and naturally occurring metal loads. Independent loading patterns in principal component analysis (PCA) provide robust evidence for distinguishing anthropogenic versus geogenic origins [17]. The inclusion of Mg in the present study, therefore, has two purposes: establishing the natural geochemical baseline and strengthening statistical source apportionment.

Fish species and study area. Three fish species occupy distinct ecological niches. *Mugil cephalus* (Flathead grey mullet) [18] is a detritivore that feeds on organic matter and surface sediments, maximising contact with particle-bound metals [19]. *Oreochromis niloticus* (Nile tilapia) [18] is an omnivorous filter-feeder that processes large volumes of water through its buccal cavity, while *Clarias gariepinus* (African sharptooth catfish) [20] is a benthic predator [21]. Feeding strategy significantly modulates metal bioaccumulation in tropical freshwater fish [22], yet species-specific data remain scarce for West African systems impacted by point-source pollution.

The Niger Delta region of Nigeria supports extensive artisanal fisheries that provide protein and livelihoods for millions, while simultaneously facing multiple anthropogenic pressures: urban runoff, industrial discharge, and artisanal refining activities [23,24]. Slaughterhouse operations add another dimension to this pollution burden, but their specific contribution to metal contamination in commercially harvested fish has not been isolated from background inputs. The inclusion of Iwofe River, a hydrologically comparable water body with no upstream abattoir operations, provides a robust baseline for establishing causal links between slaughterhouse wastewater discharge and elevated metal concentrations in fish tissues—an approach recommended by Giri and Singh [25] but rarely implemented in African field studies.

Knowledge gap and novelty. While several studies have reported metal concentrations in fish from Nigerian waters [23,24], few have translated these into probabilistic health risk estimates, using internationally accepted frameworks such as target hazard quotient (THQ) and hazard indices (HI) with reference doses from JECFA [13] and the USEPA [26]. Multi-metal risk assessment is essential because contaminants may act through additive or synergistic mechanisms. Following the recommendation of Pourret and Hursthouse [27], we refer to lead, cadmium, and chromium as “potentially toxic elements” (PTEs), while zinc, iron, and magnesium are retained as “essential trace metals.” Multi-metal risk assessment is essential because contaminants may act through additive or synergistic mechanisms [28]. To our knowledge, this is the first study integrating species-specific bioaccumulation patterns, geogenic tracer-based PCA for source identification, and comprehensive human health risk assessment (EDI, THQ, HI, CR) in abattoir-impacted West African freshwater systems. This integrated approach addresses a significant knowledge gap in the region.

Study objectives and hypothesis. This study therefore aims to: (i) quantify six potentially toxic elements (Zn, Pb, Fe, Mg, Cr, Cd) in three ecologically distinct fish species from two abattoir-impacted rivers (Transamadi and Mgbuosimini) and a control site (Iwofe); (ii) compare measured concentrations with FAO/WHO [11] and EU [12] permissible limits; (iii) apply two-way ANOVA to test for species-specific and location-specific effects; (iv) employ PCA with Mg as a potential geogenic tracer for source identification; and (v) calculate EDI, THQ, HI and CR using USEPA methodology [26] with JECFA [13] reference doses to evaluate non-carcinogenic and carcinogenic health risks. We hypothesise that metal concentrations and associated health risks will be significantly elevated in fish from impacted sites compared to the control, with Flathead grey mullet exhibiting the highest metal burdens due to its detritivorous feeding strategy. We further expect that PCA will reveal independent clustering of Mg, suggesting its utility as a geochemical baseline tracer, while Pb and Cd cluster together, indicating shared anthropogenic sources.

## 2. Methods

### 2.1. Description of the Study Site

**Figure 1:**
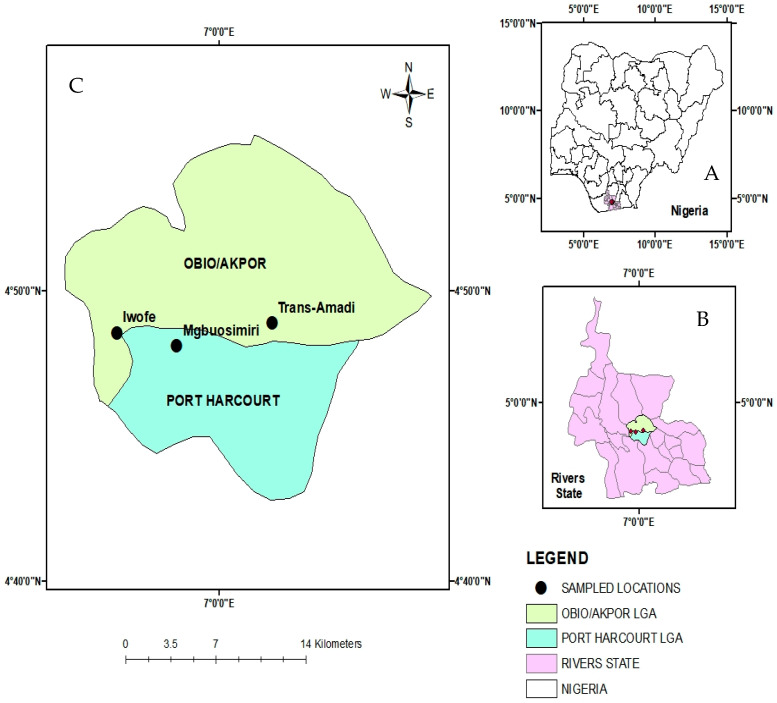
Map of the study area showing sampling locations. (**A**) Location of Nigeria in West Africa. (**B**) Rivers State showing Obio/Akpor and Port Harcourt Local Government Areas (LGAs). (**C**) The three sampling sites: Iwofe (control), Mgboshimini, and Transamadi. Red dots (●) indicate the sampling locations within Rivers State, Nigeria. The map was generated using ArcGIS Pro (Version 3.1; ESRI, Redlands, CA, USA). Approximate distances between sites are shown: Transamadi to Mgboshimini (~8 km) and Mgboshimini to Iwofe (~5 km). Scale bar: 0–14 km.

Figure 1 shows the picture view of the study site. The study site was two (2) rivers polluted by abattoir effluents located in Trans-Amadi and Mgbuosimiri (N4°48′5.43636″ (LAT); E6°58′14.53476″ (LONG); Altitude: 0fta.s.l; Orazi road, Mgbuosimiri water side/abattoir, Rivers Nigeria) and from a reference river located at Iwofe (N4°48′32.69772″ (LAT); E6°55′43.2696″ (LONG); Altitude: 33fta.s.l; Iwofe water side/abattoir, Rivers Nigeria), all in Rivers State, Nigeria. Trans-Amadi is a thousand-hectare (2500-acre) industrial area, as well as a diverse residential neighbourhood in the city of Port Harcourt. Situated at 4°48′53″ N latitude and 7°2′14″ E longitude, Trans-Amadi lies in the north and is bordered by D/line in the southwest, Woji township to the east, and Rumuola to the northwest. The main abattoir of the city is located along Trans Amadi, and Mgbuosimiri is situated in Obio-Akpor Local Government Area. The non-polluted area located at Iwofe is situated in Obio-Akpor, Rivers, Nigeria; its geographical coordinates are 4°49′4″ N latitude and 6°57′24″ E longitude. The location map (Figure 1) was generated using ArcGIS Pro (Version 3.1), a geographic information system software developed by the Environmental Systems Research Institute (ESRI, Redlands, CA, USA).

### 2.2. Sample Collection

Water and fish samples *Oreochromis niloticus* (Nile tilapia) [18], *Clarias gariepinus* (African sharptooth catfish) [20], and Flathead grey mullet (*Mugil cephalus*) (Table 1) [18] were collected during the dry season (March 2025) to minimise seasonal variability in metal concentrations. The three sampling sites were located along a 15 km stretch of the river system. Transamadi (4°48′53″ N, 7°2′14″ E) and Mgboshimini (4°48′05″ N, 6°58′14″ E) were approximately 8 km apart, while the control site Iwofe (4°49′04″ N, 6°57′24″ E) was located approximately 5 km west of Mgboshimini. Water samples were collected from multiple points (upstream, midstream, downstream) at each river site and composited to obtain a representative sample per site. These samples were analysed for physicochemical parameters (pH, conductivity, turbidity, BOD, salinity) to characterise baseline water quality and to support the interpretation of metal bioavailability.

Water quality parameters (pH, conductivity, turbidity, biochemical oxygen demand (BOD), and salinity) were analysed according to standard methods. The pH was measured using a pH meter (model HANNA H18424; Hanna Instruments, Woonsocket, RI, USA). Electrical conductivity (µS/cm) was determined using a portable electronic conductivity meter (model Mel-V; manufacturer details not specified). BOD was determined using the 5-day incubation (20 °C) titrimetric method (APHA, 2017). Turbidity (NTU) was measured using a turbidity meter (model HACH 2100AN; HACH Company, Loveland, CO, USA). Salinity was determined by the argentometric (Mohr) titrimetric method after sample pre-treatment (APHA, 2017).

No metal analysis was performed on water samples; the study’s primary focus is on fish tissue and human health risk. A location map is provided in Figure 1. Fish samples were randomly collected. At each site, similar-sized samples of each species were collected, cleaned, and wrapped in aluminium foil, then kept frozen in an ice pack before being transported to the laboratory for analysis. The water samples were collected at different points of each river. Additionally, the samples were collected aseptically with a 2-litre plastic keg filled to the brim and covered immediately to avoid external contamination.

### 2.3. Fish Specimen Characteristics

A total of 27 fish specimens were analysed (3 species × 3 locations × 3 biological replicates). For each species and location, three individual fish of similar size were purchased directly from local fishermen at riverbank landing sites adjacent to Iwofe, Mgboshimini, and Transamadi. All specimens were already dead at the point of purchase, having been caught by the fishermen using gill nets and cast nets during routine fishing activities. No live animals were handled by the researchers. The fish species sampled are listed in Table 1.

All fish specimens were measured for total length (from snout to tail fin) and weighed using a digital scale (±1 g) before tissue dissection. The mean weight and total length for each species were as follows: Nile tilapia (245 ± 32 g, 22.5 ± 1.8 cm), African sharptooth catfish (310 ± 45 g, 35.2 ± 2.1 cm), and Flathead grey mullet (180 ± 28 g, 28.5 ± 1.5 cm). Sex was not determined, as the study focused on human consumption risk, where muscle tissue is the relevant matrix regardless of sex. Only the dorsal muscle tissue (skinless fillet) was excised from each specimen for metal analysis, as they do not alter the interpretation of dietary exposure risk for consumers. This is because the primary objective was to assess human health risk from fish consumption, and we focused on the edible dorsal muscle (skinless fillet). All fish were adults of similar size to minimise size-dependent variation in metal accumulation. The raw triplicate metal concentration data for each species and location are provided in Table S1.

### 2.4. Trace Metal Analysis

In this study, potentially toxic elements (Zn, Pb, Fe, Mg, Cr, and Cd) in fish tissues were determined using atomic absorption spectrophotometry (AAS) (ContrAA 300; Analytik Jena AG, Jena, Germany). Before analysis, the samples were digested using a mixture of concentrated nitric acid under controlled conditions.

Food sample preparation/pretreatment: The fish samples were washed three times with distilled water, then the fish flesh was collected and oven-dried under low heat at 45 °C for 48 hours using a Memmert drying oven. The dried samples were homogenised (ground to powder) using a laboratory ceramic mortar and pestle. The homogenised samples were sieved with a 2 mm sieve. The homogenised samples were sieved through a 2 mm sieve to obtain a uniform particle size for digestion.

Fish sample preparation and analysis: The dried fish samples, after constant weight, were ready for metal digestion. Briefly, 5 g of each sample was weighed into a clean, dry silica dish, covered with a loose-fitting lid, and ignited in a furnace at 500 °C for 6 h until a grey-white ash was obtained. The lid was then removed to allow any residual gases to escape. After cooling, 5 mL of 10% HCl was added to enhance dissolution, followed by 5 mL of 10% HNO_3_, and the mixture was heated on a water bath until complete dissolution. The solution was transferred into a clean, dry 50 mL standard volumetric flask and made up to the mark with distilled water. The samples were accurately labelled and set for atomic absorption spectrometry (AAS) [29]. Although dry ashing can lead to the loss of some volatile elements, the target metals in this study (Pb, Cd, Cr, Zn, Fe, Mg) are stable at 500 °C under the controlled conditions used (covered crucible with loose lid). This method has been successfully applied in similar studies [29].

Atomic Absorption Spectrophotometric Analysis of Metals: Metal concentrations in digested fish muscle filtrates were determined using atomic absorption spectrophotometry (AAS) (ContrAA 300, Analytik Jena, Germany) following the method of Onyedikachi et al. [29]. Calibration standards were prepared from mono-element certified reference solutions (Inductively Coupled Plasma Standard; Merck KGaA, Darmstadt, Germany)… Sample extracts were analysed at element-specific resonance wavelengths to ensure optimal sensitivity: Cd (228.80 nm), Pb (283.30 nm), Zn (213.90 nm), Fe (248.30 nm), Mg (285.20 nm), and Cr (357.90 nm). Zinc, iron, magnesium, and chromium were analysed using hollow cathode lamps (HCL) with an air–acetylene flame atomiser, while lead and cadmium were determined using electrodeless discharge lamps (EDL) coupled with a graphite furnace atomiser to enhance sensitivity and achieve lower detection limits. Furthermore, the analytical accuracy was ensured through the following quality control measures: (i) reagent blanks were analysed with each batch to monitor contamination; (ii) calibration standards were prepared from mono-element certified reference solutions (Inductively Coupled Plasma Standard, Merck, Germany) traceable to international standards; (iii) matrix spike recovery experiments were performed for each metal, with recoveries ranging from 80% to 120%; and (iv) all samples were analysed in triplicate. These procedures follow established protocols for AAS analysis in resource-limited settings where CRMs are not always available [29].

Metal concentrations in digested fish muscle filtrates were determined using atomic absorption spectrophotometry (AAS) (ContrAA 300; Analytik Jena AG, Jena, Germany)… Zinc, iron, magnesium, and chromium were analysed using hollow cathode lamps (HCL; Analytik Jena AG, Jena, Germany) with an air–acetylene flame atomiser, while lead and cadmium were determined using electrodeless discharge lamps (EDL; Analytik Jena AG, Jena, Germany) coupled with a graphite furnace atomiser … calibration standards were prepared from mono-element certified reference solutions (Inductively Coupled Plasma Standard; Merck KGaA, Darmstadt, Germany).

All samples were analysed in triplicate, and precision was assessed using duplicate analysis with a relative per cent difference (RPD) < 10%. Calibration curves with high correlation coefficients (R^2^ ≥ 0.99) were used for quantification. Results were expressed as mean ± standard deviation in mg/kg wet weight [29].

Calculations: The potential health risks associated with fish consumption were evaluated using standard indices.

Estimated Daily Intake (EDI):

(1) EDI+C ×IR/BW

where

*C* = concentration of potentially toxic elements in fish species (*Oreochromis niloticus*, *Clarias gariepinus*, Flathead grey mullet mg/kg)

*IR* = ingestion rate of fish (0.048 kg/day)

*BW* = average body weight (70 kg)

Target Hazard Quotient (*THQ*):

(2) THQ+EDI/RFD

where:

*RFD* = Specified oral reference dose (mg/kg/day)

*THQ* values less than (<) 1 indicate no significant non-carcinogenic risk, while values greater than (>) 1 suggest a potential health concern.

Hazard Index (*HI*):

(3) HI=εTHQi

The *HI* represents the cumulative risk from multiple metals. Values above (>) 1 indicate potential health risk, while vice versa is (<).

Carcinogenic Risk (*CR*):

(4) CR=EDI×CSF

where:

*CSF* = cancer slope factor

*CR* values within 10^−6^ to 10^−4^ are considered acceptable, while higher values indicate elevated cancer risk.

The daily fish ingestion rate (*IR*) was assumed to be 0.048 kg/day (48 g/day). This value is derived from the most authoritative national per capita fish consumption figure for Nigeria of 13.3 kg/year (36 g/day) [30,31]. This is because the study area (Rivers State) is located in the coastal South-South region, where fish consumption is known to be higher than the national average (fish can provide up to 80% of animal protein intake in coastal communities) [30]. We applied a modest upward adjustment of 48 g/day. This adjusted figure remains substantially below the consumption rates reported for even non-fishing households (127 g/day) in Nigerian fishing villages [32,33], thereby ensuring a health-protective (conservative) estimate for the general adult population of Port Harcourt. This practice aligns with standard risk assessment methodology, as exemplified by the US EPA’s use of a moderate 90th percentile consumption rate (17.5 g/day) for the general population and higher percentiles only for subsistence fishers [34]. Detailed AAS instrument parameters, including wavelength, lamp current, flame type, slit width, and detection limits for each metal, are provided in File S1.

### 2.5. Assurance and Quality Control (QA/QC)

All analytical procedures were conducted under strict quality control conditions. Glassware and containers were acid-washed before use. Analytical blanks and standard reference materials were included to ensure accuracy and precision. Calibration curves with high correlation coefficients (R^2^ ≥ 0.99) were used for quantification. Each fish sample was digested and analysed in triplicate (three analytical replicates per sample). Matrix spike recovery experiments were performed in triplicate at three concentration levels (low, medium, high) for each metal. Recoveries for all metals fell within the acceptable range of 80–120%, with mean values as follows: Pb 92% (range 88–96%), Cd 88% (85–92%), Zn 105% (102–108%), Fe 96% (93–99%), Mg 102% (99–105%), and Cr 86% (83–89%). Instrument detection limits (LOD) and quantification limits (LOQ) for each metal were determined using the standard deviation of multiple blank measurements (3σ for LOD, 10σ for LOQ). The values are: Pb (LOD 0.002, LOQ 0.006 mg/kg), Cd (0.001, 0.003 mg/kg), Zn (0.005, 0.015 mg/kg), Fe (0.010, 0.030 mg/kg), Mg (0.010, 0.030 mg/kg), Cr (0.005, 0.015 mg/kg).

### 2.6. Statistical Analysis

All data were expressed as mean ± standard deviation. Statistical analyses were performed using R software (version 4.5.1; R Core Team, 2025; https://www.R-project.org/). Two-way analysis of variance (ANOVA) was applied to evaluate the effects of species and sampling location on metal concentrations. Where significant differences were observed, Tukey’s Honestly Significant Difference (HSD) test was used for post hoc comparisons. Statistical significance was set at *p* < 0.05. Assumptions of normality and homogeneity of variance were assessed before analysis. Principal component analysis (PCA) was employed to identify patterns and relationships among trace metals in fish tissues [35]. Data were standardised using z-score transformation before analysis to eliminate scale effects. Components with eigenvalues greater than 1 were retained, and factor loadings were used to interpret dominant variables contributing to observed patterns. Finally, Pearson correlation analysis was conducted to assess the relationships between variables, while hierarchical cluster analysis was applied to classify sampling locations based on similarity.

## 3. Results

### 3.1. Water Quality Parameters

The physicochemical characteristics of water from the three sites are summarised in Table 2. pH values ranged from 4.51 ± 0.03 (Mgboshimini) to 4.65 ± 0.04 (Iwofe), well below the WHO recommended range of 6.5–8.5, indicating acidic conditions across all sites.

Such high conductivity indicates elevated ionic strength, which promotes the formation of chloro-complexes (e.g., PbCl^+^, CdCl^+^), enhancing metal persistence and bioavailability. The extremely low pH favours the solubility of lead and cadmium, converting them into free ionic forms that are readily taken up by aquatic organisms.

Conductivity was extremely high at all locations (40,800–54,900 µS/cm), far exceeding the WHO guideline of 1000 µS/cm. Similarly, salinity levels (10,890–13,035 mg/L) were substantially above the WHO limit of 500 mg/L. In contrast, turbidity (0.24–0.89 NTU) and biochemical oxygen demand (BOD; 2.00–2.36 mg/L) remained within acceptable limits. These data are presented for descriptive purposes to provide context for metal bioavailability; they are not intended for statistical trend analysis due to the limited number of sampling points.

### 3.2. Water Quality Index (WQI)

To provide an integrated assessment of water quality, the weighted arithmetic Water Quality Index (WQI) was calculated following [36]. The WQI scores and classifications are presented in Table 3. Mgboshimini and Iwofe fell into the “poor” water quality category (WQI 76–100), while Transamadi was classified as “medium” (WQI 51–75). The WQI values (85.2 for Mgboshimini, 78.4 for Iwofe, and 52.1 for Transamadi) indicate that Mgboshimini has the poorest water quality among the three sites, followed by Iwofe, while Transamadi has comparatively better (medium) water quality. This pattern is consistent with the elevated lead and cadmium concentrations observed in fish from Mgboshimini and aligns with studies showing that poor water quality enhances metal bioavailability and bioaccumulation [37,38]. The WQI approach is particularly suitable here because it summarises multiple parameters into an intuitive, interpretable index, avoiding the over-interpretation of principal component analysis with only five variables. Thus, the WQI supports the link between water quality and metal contamination and provides a robust, literature-based tool for future monitoring. Additional water quality analyses, including principal component analysis eigenvalues (Table S8), loadings (Table S9), and a correlation matrix (Table S10), are available in the Supplementary Materials.

### 3.3. Physical and Chemical Description of the Testing Areas3.4. Trace Metal Bioaccumulation in Fish Tissues

LOQ = Limit of Quantification. Values below LOQ are reported as “<LOQ”. LOQ for each metal: Pb = 0.006 mg/kg, Cd = 0.003 mg/kg, Cr = 0.015 mg/kg, Zn = 0.015 mg/kg, Fe = 0.030 mg/kg, Mg = 0.030 mg/kg. Values are mean ± standard deviation (n = 3). Different superscript letters (a–c) within the same column show that there are real differences (*p* < 0.05) between locations and species based on a two-way ANOVA test followed by follow-up comparisons.

As shown in Table 4, heavy metal concentrations in fish [Nile tilapia (*Oreochromis niloticus*), African sharptooth catfish (*Clarias gariepinus*), Flathead grey mullet (*Mugil cephalus*)] varied significantly across species and sampling locations (*p* < 0.001 for all metals), demonstrating that both ecological habitat and species-specific characteristics strongly influence bioaccumulation patterns. Notably, Flathead grey mullet from Mgboshimini exhibited the highest levels of lead (1.50 ± 0.05 mg/kg) and cadmium (0.41 ± 0.02 mg/kg), exceeding both FAO/WHO permissible limits and stricter European Union standards, indicating a localised pollution concern [32]. Zinc and iron concentrations varied somewhat between species and locales, while magnesium concentrations (9.5–71.0 mg/kg) were consistently greater than those of other metals across all sites, indicating their natural geogenic origin. Zinc was highest in African sharptooth catfish from Mgboshimini (0.70 ± 0.04 mg/kg) but lowest in Nile tilapia from Iwofe and Mgboshimini (0.01 ± 0.00 mg/kg). Iron concentrations peaked in Nile tilapia from Mgboshimini (0.28 ± 0.02 mg/kg), whereas Flathead grey mullet generally showed lower iron levels. Chromium levels were low or undetectable in several samples, particularly from Transamadi and Mgboshimini, although slightly elevated in African sharptooth catfish. Cadmium showed the greatest variability, with markedly elevated levels in Flathead grey mullet from Mgboshimini, raising food safety concerns. The fish from Mgboshimini in this study consistently accumulated higher metal burdens, highlighting the impact of abattoir wastewater discharge, while interspecies differences further suggest varying capacities for metal uptake and retention.

### 3.4. Species and Location Interaction Effects

Two-way analysis of variance (ANOVA) revealed that both fish species and sampling location had highly significant effects (*p* < 0.001) on the concentration of each metal accumulated in fish tissues (Table 5). The F-values were notably high for iron (F = 1769.45) and lead (F = 1109.95), confirming that the observed differences were driven by environmental conditions rather than random variation. Thus, this combined effect was most obvious in Flathead grey mullet, where metal levels went up sharply at Mgboshimini compared to Iwofe and Transamadi, which backs up the idea that the level of pollution at a given location plays a big role in how much metal builds up in the fish. Full ANOVA outputs, including sum of squares, mean squares, F-values, and degrees of freedom, are provided in Table S3.

### 3.5. Location-Specific Metal Accumulation

**Figure 2:**
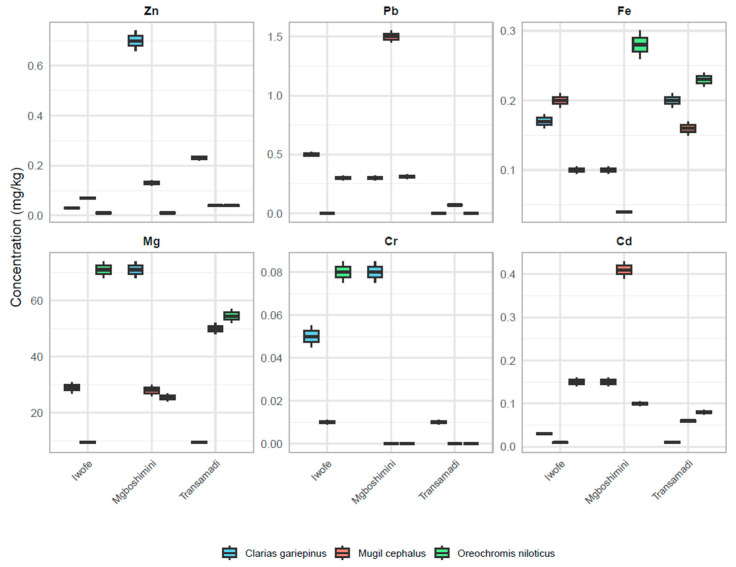
Box plots showing trace metal concentrations (mg/kg) in three fish species across three locations in Port Harcourt, Nigeria. Panels represent zinc (Zn), lead (Pb), iron (Fe), magnesium (Mg), chromium (Cr), and cadmium (Cd), respectively. Data are shown for *Clarias gariepinus* (catfish), *Mugus cephalus* (mullet), and *Oreochromis niloticus* (tilapia) collected from Iwofe (control), Mgboshimini, and Transamadi. Vertical lines indicate standard deviation, and colours distinguish location-specific differences.

As shown in Figure 2, Mgboshimini emerged as the location with the highest toxic metal accumulation. Lead reached a peak of 1.50 ± 0.05 mg/kg in Flathead grey mullet from Mgboshimini, while cadmium peaked at 0.41 ± 0.02 mg/kg in the same species. In contrast, chromium was below detectable limits in several fish species from Transamadi (Figure 2). Magnesium exhibited the highest overall concentrations across all locations, ranging from 9.5 to 71.0 mg/kg, reflecting its essential biological role and natural abundance in aquatic systems. Despite this, magnesium levels differed significantly by location (F = 171.70, *p* < 0.001). Two way analysis of variance (ANOVA) revealed that both fish species and sampling location had highly significant effects (p* < 0.001) on the concentration of each metal accumulated in fish tissues (Table 5).

Zinc (Zn): Zinc concentrations ranged from 0.01 to 0.70 mg/kg across all species and locations. While zinc is an essential micronutrient, excessive intake can be harmful. The levels detected in this study were well below the FAO/WHO permissible limit (40 mg/kg) [39], indicating no significant risk of zinc-related toxicity. Two-way ANOVA confirmed significant differences in zinc accumulation across both species and locations (*p* < 0.001), with variation attributable to species-specific feeding ecology and habitat.

Lead (Pb): Lead concentrations ranged from below detection to 1.50 ± 0.05 mg/kg, with Flathead grey mullet from Mgboshimini exhibiting the highest levels. Lead is a non-essential, potentially toxic metal with no known biological function, and even low-level exposure is associated with adverse health effects. The elevated lead levels at Mgboshimini are likely attributable to nearby industrial waste discharge, urban runoff, and abattoir-related activities, including tyre and wood combustion.

Cadmium (Cd): Cadmium was detected only in select samples, with concentrations ranging from 0.01 to 0.41 mg/kg. The highest levels were found in Flathead grey mullet from Mgboshimini (0.41 ± 0.02 mg/kg). Cadmium is highly toxic and bioaccumulative, with a long biological half-life in humans. Its detection only at specific locations suggests a point-source origin, most likely industrial effluent discharge and abattoir waste. Although concentrations were low at most sites, regular monitoring remains essential.

Iron (Fe): Iron concentrations ranged from 0.04 to 0.28 mg/kg, with the highest levels observed in Nile tilapia from Mgboshimini (0.28 ± 0.02 mg/kg). Iron is an essential element required for haemoglobin synthesis and enzymatic functions. However, excessive iron intake can induce oxidative stress and cellular damage. The elevated iron levels at Mgboshimini may likely originate from natural soil weathering, rusting infrastructure, or abattoir blood discharge [40]. All recorded values were within acceptable safety ranges.

Mgboshimini consistently exhibited higher concentrations of most metals compared to Iwofe (control) and Transamadi, indicating greater anthropogenic pollution at that location. While most metal levels remained below international safety thresholds, the elevated lead and cadmium concentrations at Mgboshimini, particularly in Flathead grey mullet, exceeded permissible limits (FAO/WHO: Pb 0.5 mg/kg, Cd 0.5 mg/kg; EU: Cd 0.05 mg/kg), signalling potential public health concerns. Regular monitoring and source control measures are therefore recommended.

### 3.6. Multivariate Analysis of Metal Interaction and Correlation

**Figure 3:**
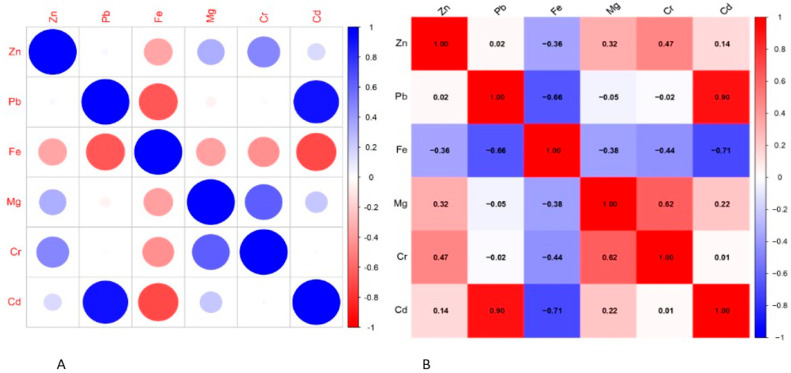
Multivariate analysis. (**A**) Correlation map showing how the potentially toxic elements (zinc, lead, iron, magnesium, chromium, and cadmium) relate to each other across the fish species and locations tested. Colours indicate correlation strength, ranging from −1 (strong negative, blue) to +1 (strong positive, red). Lead and cadmium were strongly correlated (r = 0.90), as were magnesium and chromium (r = 0.62). Iron showed negative correlations with cadmium (r = −0.71) and lead (r = −0.66). (**B**) Correlation chart showing relationships among Zn, Pb, Fe, Mg, Cr, and Cd.

Correlation analysis [41] revealed distinct relationships among the measured metals, indicating both shared sources and divergent environmental behaviours (Figure 3A). A very strong positive correlation was observed between lead and cadmium (r = 0.90), suggesting their co-occurrence and likely origin from common anthropogenic inputs such as industrial effluents or contaminated sediments. Magnesium and chromium exhibited a moderate positive correlation (r = 0.62), which may reflect geogenic influences, particularly the co-deposition of mineral-rich sediments. In contrast, iron showed consistent negative correlations with cadmium (r = −0.71), lead (r = −0.66), and chromium (r = −0.44), indicating that it follows a different environmental pathway, possibly influenced by variations in sediment composition, redox conditions, and pH-dependent mobility. Several metal pairs, including lead and magnesium (r = −0.05), chromium and cadmium (r = 0.01), and zinc and lead (r = 0.02), exhibited negligible correlations, implying no meaningful association in their distribution patterns. These findings highlight that while certain metals, particularly lead and cadmium, may likely originate from similar pollution sources, others, such as iron, behave independently, underscoring the complexity of metal dynamics in aquatic systems. Importantly, these correlations indicate associations rather than causation and should be interpreted as evidence of possible common sources or environmental controls rather than direct mechanistic relationships. The complete Pearson correlation matrix for all metal pairs is presented in Table S4.

As shown in Figure 3B, correlation coefficients ranged from strongly negative to strongly positive, indicating diverse interrelationships among the metals and reflecting differences in their sources and environmental behaviour. Lead and cadmium exhibited a very strong positive correlation (r ≈ 0.90), demonstrating a consistent co-occurrence and suggesting a common anthropogenic origin. Magnesium showed a moderate positive relationship with chromium (r ≈ 0.62), while chromium also correlated moderately with zinc (r ≈ 0.47), indicating possible shared geochemical influences. In contrast, iron displayed inverse relationships with several metals, including cadmium (r ≈ −0.71) and lead (r ≈ −0.66), as well as a moderate negative correlation with chromium (r ≈ −0.44), suggesting differing environmental controls such as redox conditions or sediment interactions. Weaker associations were also observed, with zinc showing a slight positive correlation with magnesium (r ≈ 0.32) and a slight negative correlation with iron (r ≈ −0.36). Consequently, these patterns highlight clear clustering, particularly between lead and cadmium, alongside a distinctly different behaviour of iron compared to the other metals, underscoring the complexity of metal dynamics in the aquatic environment.

### 3.7. Principal Component Analysis of Location-Specific Metal Accumulation

**Figure 4:**
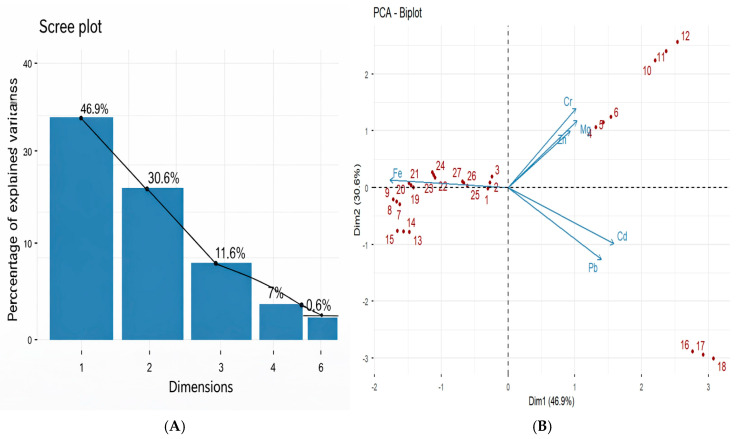
Principal component analysis. (**A**) Scree plot showing the distribution of explained variance across principal components. (**B**) Biplot of principal component analysis showing relationships among metal variables and samples. Principal component analysis loadings and eigenvalues for all six components are provided in Table S5.

The scree plot [42] as seen in Figure 4A illustrates the distribution of explained variance across the principal components, showing a steady decline as component number increases and indicating that only a few components capture most of the meaningful information in the dataset. The first principal component (PC1) accounted for 46.9% of the total variance, followed by PC2 (30.6%) and PC3 (11.6%), while subsequent components contributed minimally (PC4: 7.0%, PC5: 3.3%, PC6: 0.6%). Cumulatively, the first three components explained 89.1% of the total variance, demonstrating that the majority of data variability can be effectively represented within these dimensions. A clear inflexion point (elbow) at PC3 further confirms that additional components contribute little new information. Furthermore, the PCA biplot (Figure 4B), based on PC1 and PC2, which together explain 77.5% of the variance, effectively visualises the relationships among samples and metal variables. Loadings indicate that chromium (Cr) and magnesium (Mg) are strongly aligned in the positive direction along PC1, driving variability in samples with high PC1 scores, whereas iron (Fe) loads negatively, reflecting contrasting behaviour. In contrast, cadmium (Cd) and lead (Pb) are primarily associated with PC2, with their strong loadings indicating a major role in vertical sample separation. Accordingly, samples positioned on the positive side of PC1 are influenced by Cr and Mg, those on the negative side by Fe, while variation along PC2 distinguishes samples with elevated Pb and Cd concentrations.

### 3.8. Human Health Risk from Potentially Toxic Elements in Fish Species

Health risk assessments [43], involving the estimated daily intake (EDI), target hazard quotient (THQ), hazard index (HI), and cancer risk (CR) values for potentially toxic elements across fish species and sampling locations (Table 6), revealed important patterns in both non-carcinogenic and carcinogenic risk. Complete health risk indices for each metal and each species-location combination are presented in Table S6, and a summary of hazard index and total cancer risk is provided in Table S7. This revealed important patterns in both non-carcinogenic and carcinogenic risk. THQ values for individual metals were generally below the safety threshold of 1, indicating that exposure to single metals is unlikely to pose significant non-cancer health risks; however, relatively elevated THQ values were observed for lead (Pb = 0.884) and cadmium (Cd = 0.966) in Flathead grey mullet from Mgboshimini, approaching critical levels. More notably, cumulative risk assessment showed that HI values exceeded 1 in several cases, including *Nile tilapia* from Iwofe (HI = 1.151), *African sharptooth catfish* from Mgboshimini (HI = 1.157), and Flathead grey mullet from Mgboshimini (HI = 2.072), indicating potential health concerns due to combined metal exposure despite individually acceptable levels. Cancer risk values for Pb, Cr, and Cd ranged between 10^−6^ and 10^−4^, within acceptable limits, but with some approaching the upper threshold, particularly in Flathead grey mullet from Mgboshimini (3.97 × 10^−4^) and Nile tilapia from Iwofe (2.35 × 10^−4^), suggesting possible long-term carcinogenic risk with sustained consumption. Spatial variation showed Mgboshimini as the highest risk area, likely influenced by anthropogenic activities, followed by Iwofe with moderate risk, while Transamadi exhibited comparatively lower risk levels. Species-specific differences were also evident, with Flathead grey mullet consistently demonstrating higher THQ, HI, and CR values, indicating a greater capacity for metal accumulation, while Nile tilapia also contributed notably to dietary exposure in certain locations. Generally, these findings underscore the importance of considering both cumulative exposure and species-specific bioaccumulation in assessing public health risks associated with fish consumption.

### 3.9. Multi-Factor Risk Modelling and Human Health Assessment

**Figure 5:**
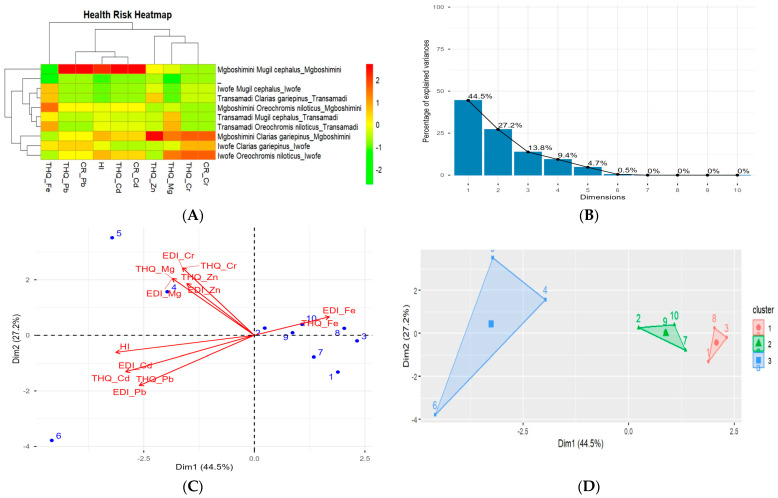
Multivariate analysis of health risk indices. (**A**) Hierarchical cluster analysis heatmap of health risk levels. Rows represent species-location combinations; columns represent risk measures (THQ: Target Hazard Quotient; HI: Hazard Index; CR: Cancer Risk). (**B**) Scree plot of explained variance. (**C**) PCA biplot of health risk indices. (**D**) K-means cluster plot showing species-location groupings based on health risk profiles.

Fish species, sample sites, and many health risk indices clearly interacted, according to the cluster analysis heat map (Figure 5A), which is based on standardised scores where red represents high risk and green suggests safe levels [44]. Hierarchical clustering grouped the risk metrics into two primary categories: non-carcinogenic risks (THQ and HI) and carcinogenic risks (CR), reflecting distinct but related exposure patterns. The heat map highlighted Flathead grey mullet from Mgboshimini as the most critical risk group, exhibiting intense red colouration across key indicators, including THQ_Pb, CR_Pb, HI, THQ_Cd, and CR_Cd, signifying consistently elevated risk levels. A secondary high-risk cluster was observed for African sharptooth catfish from Mgboshimini, particularly associated with THQ_Zn, THQ_Cr, and CR_Cr. In contrast, Nile tilapia and Flathead grey mullet from Transamadi and Iwofe displayed predominantly green shades, indicating comparatively safe exposure levels. Further multivariate analysis using principal component analysis (PCA) and K-means clustering supported these findings. The scree plot (Figure 5B) showed that the first two dimensions explained 71.7% of total variance (44.5% and 27.2%), providing a robust representation of the dataset. The PCA biplot (Figure 5C) revealed a strong clustering of HI, THQ_Pb, EDI_Pb, THQ_Cd, and EDI_Cd along the negative axis of Dim1, identifying Pb and Cd as the primary drivers of non-carcinogenic risk, while essential elements such as Fe, Zn, and Mg formed a separate cluster, reflecting distinct behaviour. The K-means cluster plot (Figure 5D) further distinguished three groups, with Flathead grey mullet from Mgboshimini forming a distinct outlier, positioned along the negative axis of Dim1 and closely aligned with HI and THQ_Pb, confirming it as the sample with the highest overall health risk in the study. To account for uncertainty arising from the limited sample size (n = 3 per species per location), the hazard index for Flathead grey mullet at Mgboshimini was recalculated using the mean ± standard deviation of metal concentrations. The resulting HI ranged from 1.89 to 2.25 (mean 2.07), with the lower bound still exceeding the safety threshold of 1.0. This indicates that the finding of potential health risk is robust despite the small sample size.

Uncertainty analysis due to limited sample size. To assess the robustness of the health risk estimates given the small sample size (n = three per species per location), we recalculated the hazard index (HI) for the species-location combination with the highest risk (Flathead grey mullet at Mgboshimini) using the mean ± standard deviation of each metal concentration. The resulting HI values ranged from 1.89 to 2.25 (mean 2.07). The lower bound of this range (1.89) still exceeds the safety threshold of 1.0, indicating that the finding of potential non-carcinogenic health risk is robust despite the limited sample size. For other species-location combinations where HI exceeded 1.0 (Nile tilapia at Iwofe and African sharptooth catfish at Mgboshimini), similar calculations confirmed that the minimum HI remained above 1.0.

## 4. Discussion

### 4.1. Water Quality as a Driver of Metal Bioavailability

In this study, the physicochemical characteristics of water from Iwofe, Transamadi, and Mgboshimini indicate a system under significant contamination stress, with consistently acidic pH values (4.51–4.65) falling well below the recommended World Health Organisation range (6.5–8.5). While pH alone is not a direct indicator of pollution, it critically influences metal solubility and mobility, as acidic conditions favour the presence of metals such as lead (Pb) and cadmium (Cd) in their free ionic forms, thereby enhancing their bioavailability and uptake by aquatic organisms [45]. The narrow pH range across all sites suggests sustained acidifying inputs, likely driven by continuous discharge of abattoir effluents, where microbial decomposition of organic waste releases organic acids and carbon dioxide [46]. Such conditions not only promote metal desorption from sediment binding sites through proton competition but also disrupt ionoregulatory balance in fish, impairing sodium and calcium transport, inducing oxidative stress, and increasing vulnerability to metal toxicity. In addition, conductivity, turbidity, and salinity were markedly elevated beyond permissible limits, reflecting high ionic loads associated with dissolved salts, organic residues, and mineralised waste typical of abattoir discharges [47]. Elevated turbidity indicates substantial suspended particulate matter, which can act as carriers for metals, while high ionic strength enhances metal persistence through the formation of chloro-complexes (e.g., PbCl^+^, CdCl^+^) and imposes osmotic stress on aquatic organisms, further facilitating bioaccumulation [48]. Although biochemical oxygen demand (BOD) values were only moderately elevated, suggesting non-excessive organic loading, this may mask underlying risks, as dissolved contaminants and ionic interactions often drive toxicity more than conventional physical parameters. These results emphasise the necessity for integrated assessment approaches in assessing heavy metal pollution in wastewater-impacted systems, since relying just on standard water quality indicators may underestimate ecological risk [24]. The observed low pH and high conductivity at Mgboshimini are consistent with conditions that favour metal solubility, which may explain the elevated Pb and Cd levels in fish from this site.

The combined presence of acidic pH, elevated conductivity, high turbidity, and increased salinity indicates a system predominantly impacted by chemical pollution rather than solely organic or particulate loading. Such conditions enhance the mobilisation, sequestration, and subsequent accumulation of metals from sediments into the water column, facilitating their transfer into aquatic organisms and ultimately into humans through dietary exposure. The consistent deviation of these parameters across all sampling sites strongly suggests that anthropogenic activities are the primary drivers of water quality deterioration, with continuous inputs of potentially toxic elements and chemical leachates sustaining contamination beyond natural baseline levels. This widespread and uniform pattern reflects persistent external influence rather than isolated events. Supporting this, heat map analysis revealed spatial clustering of elevated conductivity and biochemical oxygen demand (BOD) at specific locations, highlighting zones of intensified chemical loading typical of wastewater-impacted environments where localised discharges create distinct contamination gradients. These conditions compromise the suitability of the water for drinking, irrigation, and aquatic life, while also posing significant risks of food chain contamination and human health exposure [49,50].

### 4.2. Water Quality Index and Links to Metal Bioavailability

The Water Quality Index (WQI) provides an integrated, interpretable summary of the physicochemical conditions across the three sites. Mgboshimini and Iwofe fell into the “poor” water quality category (WQI 85.2 and 78.4), while Transamadi was classified as “medium” (52.1). This classification is supported by the individual parameters: all three sites exhibited strongly acidic pH (4.51–4.65), far below the WHO-recommended range of 6.5–8.5. Acidic conditions favour the solubility of lead and cadmium, converting them into free ionic forms that are readily taken up by aquatic organisms [45]. High conductivity (40,800–54,900 µS/cm) and salinity (10,890–13,035 mg/L) indicate elevated ionic strength, which promotes the formation of chloro-complexes (e.g., PbCl^+^, CdCl^+^), enhancing metal persistence and bioavailability in the water column [48]. Elevated turbidity at Mgboshimini (0.89 NTU) points to suspended particulate matter that can act as a carrier for metals into benthic sediments.

The poorest water quality at Mgboshimini (WQI 85.2) coincides with the highest concentrations of Pb and Cd in fish from that site, particularly in the detritivorous Flathead grey mullet (Mugil cephalus). This spatial concordance strongly indicates that degraded water quality enhances metal mobilisation, sediment loading, and subsequent bioaccumulation in bottom-feeding fish. In contrast, Transamadi’s medium water quality (WQI 52.1) is associated with lower metal burdens across all species, reinforcing the link between water quality and human health risk. These patterns align with recent findings from Nigerian water bodies, where poor water quality was similarly associated with elevated metal levels in fish [37,38]. The WQI evidence demonstrates that slaughterhouse-impacted sites experience physicochemical degradation that directly promotes metal bioavailability and uptake by aquatic organisms, indicating their roles in species-specific bioaccumulation patterns discussed below.

### 4.3. Species-Specific Bioaccumulation and Source Discrimination

Metal concentrations in fish tissues varied significantly by species and location (*p* < 0.001). Flathead grey mullet (*Mugil cephalus*) from Mgboshimini accumulated the highest levels of lead (1.50 ± 0.05 mg/kg) and cadmium (0.41 ± 0.02 mg/kg), exceeding EU maximum limits for fish muscle (Pb 0.30 mg/kg, Cd 0.05 mg/kg) by 500% and 800%, respectively. This striking enrichment reflects the species’ detritivorous feeding ecology: mullet constantly ingests organic detritus and surface sediments where metals preferentially accumulate under the acidic, ion-rich conditions documented at this site [19]. In contrast, Nile tilapia (*Oreochromis niloticus*) and African sharptooth catfish (*Clarias gariepinus*)—an omnivorous filter-feeder and a benthic predator—exhibited substantially lower metal burdens, consistent with reduced direct contact with contaminated sediment [24]. Although detritivorous species generally occupy a lower trophic position, their direct sediment interaction makes them highly susceptible in contaminated environments [51,52]. This sediment-to-biota transfer pathway supports the use of M. cephalus as a sensitive bioindicator of sediment-associated pollution.

Strong positive correlation between Pb and Cd (r = 0.90) and their joint loading on the same principal component (PC2) indicate a shared anthropogenic origin. Local practices such as discharge of untreated abattoir effluent (blood, manure, offal) and combustion of scrap automobile tyres for hide singeing are the most plausible sources, as tyre combustion releases Pb and Cd as fine particulate matter [53,54]. By contrast, magnesium loaded independently on PC1 and correlated strongly with chromium (r = 0.62), suggesting a primarily geogenic origin controlled by natural geological weathering [14]. Recent high-impact studies have advanced such source apportionment: Majee et al. [15] used positive matrix factorisation (PMF) and thermodynamic modelling to quantitatively separate geogenic from anthropogenic trace-element sources in groundwater; Joe et al. [16] combined PMF with metal isotopes to track contamination in estuarine sediments. Our PCA-based approach, though simpler, follows the same principle—using multivariate analysis of fish tissue data alone to effectively distinguish anthropogenic (Pb-Cd) from natural (Mg-Cr) metal signals. This provides a cost-effective method for source identification in resource-limited settings, complementing more advanced isotopic techniques.

### 4.4. Spatial Dynamics and Anthropogenic Influence

The pronounced spatial variation strongly indicates that localised anthropogenic activities drive the contamination, with Mgboshimini as a critical hotspot for Pb and Cd. This pattern is consistent with point-source pollution from abattoir-related practices: discharge of organic waste (blood, manure, offal), combustion of scrap automobile tyres for singeing animal hides, and firewood or petroleum-assisted burning [53]. While we did not directly measure tyre combustion emissions, previous studies have shown that tyre combustion releases Pb and Cd as fine particulate matter, along with characteristic organic tracers (e.g., benzothiazoles) [54]. The presence of these metals in fish from sites where tyre singeing is routine is therefore consistent with an anthropogenic input from this source.

These processes generate a complex pollution “fingerprint” that readily associates with sediments and becomes highly bioavailable to benthic organisms such as Flathead grey mullet. Additional contributions from firewood ash and abattoir effluents further enrich sediments with potentially toxic elements, explaining the elevated burdens observed in bottom-feeding fish species [50,54]. Lower metal concentrations at Transamadi likely reflect strong tidal flushing within the Bonny River estuary, which dilutes and disperses contaminants. In contrast, limited tidal exchange at the more confined Mgboshimini site promotes pollutant retention and sediment enrichment, consistent with studies from similar Niger Delta systems. Correlation analysis reinforces source attribution: strong Pb-Cd associations indicate shared anthropogenic origins, while the independent behaviour of magnesium (Mg) is consistent with a primarily geogenic background role, supporting its potential use as a reference element. Iron exhibited mixed behaviour, reflecting both natural and anthropogenic inputs as well as redox-sensitive interactions [55,56], while moderate associations between chromium, zinc, and magnesium suggest combined natural and human influences. These findings highlight the complex interplay of pollution sources, hydrodynamics, and geochemical processes in shaping metal distribution and bioaccumulation.

### 4.5. Biogeochemical Drivers of Site Contamination

The Water Quality Index (WQI) and multivariate analysis of fish tissue data together elucidate the drivers of metal contamination. Mgboshimini’s poor WQI (85.2) and its strong Pb-Cd signal in fish indicate that sediment-mediated exposure is the dominant pathway. High turbidity (0.89 NTU) reflects elevated suspended particulate matter, which carries Pb and Cd adsorbed onto clay minerals, organic matter, and iron oxides. Elevated salinity promotes chloro-complex formation (e.g., PbCl^+^, CdCl^+^), enhancing metal persistence and bioavailability [48]. These conditions favour accumulation in benthic detritivores such as *M. cephalus*. By contrast, Transamadi’s medium WQI (52.1) and lower metal burdens align with effective tidal flushing and reduced sediment retention.

The independent loading of Mg in the fish-tissue PCA reinforces its role as a natural geochemical baseline. While organic inputs (indicated by BOD) may drive intermittent metal mobilisation through reductive dissolution of iron oxides, the dominant control on sustained metal exposure—particularly at Mgboshimini—is the geochemical matrix governed by turbidity and salinity. Thus, the integrated WQI-PCA evidence shows that sustained metal exposure at impacted sites is governed by the geochemical matrix (turbidity, salinity) and species feeding ecology, not by episodic organic pollution alone. This integrated approach highlights the value of combining WQI with PCA and ecological insights to elucidate metal sources, explain spatial variability, and better predict environmental and public health risks in contaminated aquatic systems [48,56].

### 4.6. Exposure and Bioaccumulation Patterns

The relatively higher estimated daily intake (EDI) values for magnesium across all species are expected due to its essential physiological role and natural abundance in aquatic systems. In contrast, elevated concentrations of lead and cadmium, especially in *M. cephalus* from Mgboshimini, raise significant environmental and public health concerns, given the reliance of local populations on fish as a primary protein source. Chronic dietary exposure to Pb and Cd has been linked to neurodevelopmental impairment in children, renal dysfunction, and increased carcinogenic risk, reinforcing the need for continuous monitoring and effective source control [57]. The elevated Pb levels observed in African sharptooth catfish and Flathead grey mullet point to strong anthropogenic inputs, likely associated with industrial discharge, petroleum-related activities, and urban runoff characteristic of the Niger Delta region. The higher cadmium accumulation in mullet suggests a strong sediment-associated pathway, as Cd readily binds to particulate matter and enters the food chain through benthic feeding [43,51,58]. This pattern aligns with the prevailing environmental conditions at impacted sites: high turbidity, salinity, and low pH, which collectively enhance metal mobility, sediment interaction, and bioavailability in aquatic ecosystems.

### 4.7. Non-Carcinogenic and Carcinogenic Risks

Individual target hazard quotients (THQ) for Pb (0.88) and Cd (0.97) in Mugil cephalus from Mgboshimini approached the safety threshold of 1.0. The cumulative hazard index (HI) exceeded 1.0 in all species from Mgboshimini, peaking at 2.07 for mullet. Uncertainty analysis using the standard deviation of metal concentrations gave an HI range of 1.89–2.25—all values above 1.0. This demonstrates that the finding of potential non-carcinogenic risk is robust despite the limited sample size (n = three per species per location).

Carcinogenic risk (CR) for M. cephalus (3.97 × 10^−4^) exceeded the upper acceptable limit of 10^−4^, driven primarily by cadmium—a known human carcinogen linked to kidney, lung, and prostate cancers. The contribution of chromium remains uncertain due to the lack of speciation between Cr (III) and the more toxic Cr (VI). Regular consumption of fish from Mgboshimini, particularly detritivorous species, therefore, poses potential long-term health risks, especially for vulnerable populations. These findings align with global trends linking industrialisation and urbanisation to increased metal contamination in aquatic food systems [59]. The elevated cadmium level (0.41 mg/kg) is especially alarming given international efforts to minimise dietary exposure, as chronic ingestion can lead to renal dysfunction. The consistently high iron levels observed across species may also contribute to oxidative stress in fish, reflecting a broader burden of mineral and metal toxicity within the aquatic environment [51,57].

### 4.8. Study Limitations

Several limitations of this study should be acknowledged when interpreting the findings.

Temporal representativeness. Sampling was restricted to a single time point during the dry season (March 2025). Metal concentrations in water and fish tissues can vary seasonally due to changes in rainfall, runoff, hydrological flow patterns, and anthropogenic activities. The dry season may concentrate pollutants through reduced dilution, potentially leading to higher measured values than would be observed during the wet season. Conversely, wet season runoff could introduce additional contaminants from wider catchment areas. Therefore, the findings presented here may not be directly generalisable to other seasons or inter-annual variation.

Spatial representativeness. The study was limited to three river locations (Iwofe, Mgboshimini, and Transamadi) within Port Harcourt, Rivers State, Nigeria. While these sites include a control location and two slaughterhouse-impacted locations, the results may not reflect conditions in other abattoir-impacted rivers across Nigeria or West Africa. Variations in wastewater volume, treatment practices, hydrodynamic conditions, and baseline water chemistry between different abattoir systems could yield different bioaccumulation patterns and health risk profiles. Extrapolation to other regions should therefore be made with caution.

Source attribution of Pb and Cd to specific anthropogenic activities (slaughterhouse effluent, tyre combustion) is based on PCA clustering, site observations, and literature, not on direct chemical analysis of effluent, water, sediment, or tyre combustion products. Future studies should measure metal concentrations and organic tracers in abattoir wastewater, suspended particulate matter, and sediment to confirm source pathways.

Sample size. A total of 27 fish specimens were analysed (three species × three locations × three biological replicates). This sample size, while sufficient for preliminary assessment and two-way ANOVA, limits statistical power to detect subtle differences and may not capture the full range of metal concentrations within each fish population. Nevertheless, the consistency of elevated lead and cadmium concentrations in Flathead grey mullet at Mgboshimini, coupled with HI values exceeding 1.0 across multiple species at this location (and the uncertainty analysis showing HI lower bound still >1.0), suggests that the observed health risks are not artefacts of limited sampling.

In addition, the age and sex of the fish were not recorded. Metal bioaccumulation can vary with age, size, and reproductive status. However, our study was designed to assess human health risk from consumption of the edible muscle; for this purpose, the use of adult fish of similar size is sufficient. Future studies may investigate age- and sex-related differences for ecological or toxicological insights.

## 5. Conclusions and Recommendations

The present study provides a comprehensive assessment of water quality and contaminant dynamics in abattoir-influenced fish species: Nile tilapia (*Oreochromis niloticus*), African sharptooth catfish (*Clarias gariepinus*), and Flathead grey mullet (*Mugil cephalus*) in Rivers State, Nigeria. The physicochemical profile revealed consistently acidic conditions and exceptionally high conductivity and salinity across all sampling locations, with values far exceeding recommended limits. These conditions indicate a chemically stressed environment in which ionic pollution dominates over conventional indicators of organic contamination, as reflected by relatively low turbidity and moderate biochemical oxygen demand. Trace metal analysis demonstrated significant spatial and species-dependent variation, with elevated concentrations observed particularly at Mgboshimini. The statistical evidence indicates that both environmental exposure and biological characteristics influence metal accumulation patterns in fish. The occurrence of lead and cadmium at elevated levels in edible tissues—especially in Flathead grey mullet from the most polluted site (Mgboshimini)—raises concerns regarding potential human exposure through dietary intake.

Additionally, the combined influence of low pH and high ionic strength may enhance the mobility and bioavailability of contaminants, thereby amplifying ecological and toxicological risks. This interaction suggests that physicochemical conditions are not merely background variables but active drivers of contaminant behaviour within the aquatic system. The observed patterns point towards sustained anthropogenic inputs, most plausibly linked to abattoir discharges and associated waste streams. The findings further suggest a system undergoing chemical alteration, with potential consequences for aquatic biodiversity, species composition, and ecosystem functioning. From a public health standpoint, the accumulation of potentially toxic elements in commonly consumed fish species indicates a possible pathway for human exposure, particularly in the Mgboshimini community, which relies heavily on local fisheries.

Despite the limitations of single-season sampling and small sample size, the consistent exceedance of safety thresholds and the uncertainty analysis support the conclusion that slaughterhouse wastewater discharge poses potential health risks to fish consumers in the study area. These findings highlight the need for targeted environmental management strategies.

Future studies should incorporate seasonal sampling, expanded spatial coverage (more sites and a larger geographical area), larger sample sizes per species and location, and probabilistic risk assessment approaches (e.g., Monte Carlo simulation) to better characterise uncertainty.

This study contributes to the growing body of evidence that freshwater systems in rapidly urbanising regions are increasingly affected by complex mixtures of pollutants. Addressing these challenges requires an integrated approach that links scientific assessment with policy implementation and community-level awareness.

## Figures and Tables

**Table 1:** List of fish species sampled from the three study sites in Rivers State, Nigeria, with their botanical and common names.

Fish Samples	Botanical Names	Common Names
	*Oreochromis niloticus*	Nile tilapia, Mango fish, Nilotica, Boulti,
	*Mugil cephalus*	Flathead mullet, grey mullet, Bully mullet
	*Clarias gariepinus*	African sharptooth catfish, Mudfish, African Catfish

**Table 2:** Physical and chemical parameters of water samples collected from three study sites.

Parameter	Iwofe	Mgboshimini	Transamadi	WHO Guideline Limit
pH	4.65 ± 0.04	4.51 ± 0.03	4.63 ± 0.01	6.5–8.5
Conductivity (µS/cm)	51,450.33 ± 1.76	54,900.00 ± 10.32	40,800.47 ± 0.65	≤1000
Turbidity (NTU)	0.24 ± 0.02	0.89 ± 0.03	0.43 ± 0.00	≤5
BOD (mg/L)	2.00 ± 0.09	2.23 ± 0.32	2.36 ± 0.06	≤3
Salinity (mg/L)	12,375.00 ± 977.82	13,035.27 ± 225.73	10,890.37 ± 35.75	≤500

Values are expressed as mean ± standard deviation. WHO guideline limits are included for comparison. All analyses were performed according to APHA standard methods. The raw triplicate measurements for each site are presented in Table S2.

**Table 3:** Water Quality Index (WQI) scores and classifications for the three sampling sites.

Site	WQI Score	Classification
Iwofe	78.4	Poor
Mgboshimini	85.2	Poor
Transamadi	52.1	Medium

WQI was calculated using the weighted arithmetic method of Brown et al. [36] based on pH, conductivity, turbidity, BOD, and salinity. Classification criteria: 0–25 = Excellent; 26–50 = Good; 51–75 = Medium; 76–100 = Poor; >100 = Very Poor.

**Table 4:** Concentrations of potentially toxic elements (mg/kg, mean ± standard deviation) in fish species across three sampling locations. Values below the limit of quantification are reported as < LOQ. Different superscript letters (a–c) within the same column indicate significant differences (*p* < 0.05) between locations and species based on two-way ANOVA followed by Tukey’s post-hoc test.

Species	Location	Zn	Pb	Fe	Mg	Cr	Cd
*Clarias gariepinus*	Iwofe	0.03 ± 0.00 ^c^	0.50 ± 0.02 ^a^	0.17 ± 0.01 ^b^	29.0 ± 2.0 ^b^	0.05 ± 0.01 ^a^	0.03 ± 0.00 ^c^
	Mgboshimini	0.70 ± 0.04 ^a^	0.30 ± 0.02 ^b^	0.10 ± 0.00 ^c^	71.0 ± 3.0 ^a^	0.08 ± 0.01 ^a^	0.15 ± 0.01 ^b^
	Transamadi	0.23 ± 0.01 ^b^	<LOQ	0.20 ± 0.01 ^a^	9.5 ± 0.5 ^c^	0.01 ± 0.00 ^b^	0.01 ± 0.00 ^c^
*Oreochromis niloticus*	Iwofe	0.01 ± 0.00 ^c^	0.30 ± 0.02 ^b^	0.10 ± 0.00 ^c^	71.0 ± 3.0 ^a^	0.08 ± 0.01 ^a^	0.15 ± 0.01 ^a^
	Mgboshimini	0.01 ± 0.00 ^c^	0.31 ± 0.02 ^b^	0.28 ± 0.02 ^a^	25.5 ± 1.5 ^b^	<LOQ	0.10 ± 0.00 ^b^
	Transamadi	0.04 ± 0.00 ^b^	<LOQ	0.23 ± 0.01 ^b^	54.5 ± 2.5 ^a^	<LOQ	0.08 ± 0.01 ^b^
*Mugil cephalus*	Iwofe	0.07 ± 0.00 ^b^	<LOQ	0.20 ± 0.01 ^b^	9.5 ± 0.5 ^c^	0.01 ± 0.00 ^b^	0.01 ± 0.00 ^c^
	Mgboshimini	0.13 ± 0.01 ^a^	1.50 ± 0.05 ^a^	0.04 ± 0.00 ^c^	28.0 ± 2.0 ^b^	<LOQ	0.41 ± 0.02 ^a^
	Transamadi	0.04 ± 0.00 ^b^	0.07 ± 0.00 ^b^	0.16 ± 0.01 ^b^	50.0 ± 2.0 ^a^	<LOQ	0.06 ± 0.00 ^b^

**Table 5:** Two-way analysis of variance (ANOVA) results showing the effects of species and location on metal concentrations in fish tissues. F-values are presented for each metal. *** = Significant at *p* < 0.001.

Metal	Species (F)	Location (F)	Significance
Zn	239.97	445.23	***
Pb	1109.95	590.70	***
Fe	563.85	1769.45	***
Mg	107.86	171.70	***
Cr	494.81	397.40	***
Cd	281.28	729.27	***

**Table 6:** Estimated Daily Intake (EDI), Target Hazard Quotient (THQ), Hazard Index (HI), and Cancer Risk (CR) associated with heavy metal exposure through consumption of selected fish species from Iwofe, Mgboshimini, and Transamadi.

A. Estimated Daily Intake (EDI) *Clarias gariepinus* (catfish), Flathead grey mullet (mul-let), and *Oreochromis niloticus* (tilapia)									
**Location**	**Species**	**EDI_Zn**	**EDI_Pb**		**EDI_Fe**	**EDI_Mg**	**EDI_Cr**		**EDI_Cd**
Iwofe	*Clarias gariepinus*	7.07 × 10^−5^	1.18 × 10^−3^		4.01 × 10^−4^	6.84 × 10^−2^	1.18 × 10^−4^		7.07 × 10^−5^
Iwofe	*Mugil cephalus*	1.65 × 10^−4^	0		4.71 × 10^−4^	2.24 × 10^−2^	2.36 × 10^−5^		2.36 × 10^−5^
Iwofe	*Oreochromis niloticus*	2.36 × 10^−5^	7.07 × 10^−4^		2.36 × 10^−4^	1.67 × 10^−1^	1.89 × 10^−4^		3.54 × 10^−4^
Mgboshimini	*Clarias gariepinus*	1.65 × 10^−3^	7.07 × 10^−4^		2.36 × 10^−4^	1.67 × 10^−1^	1.89 × 10^−4^		3.54 × 10^−4^
Mgboshimini	*Mugil cephalus*	3.06 × 10^−4^	3.54 × 10^−3^		9.43 × 10^−5^	6.60 × 10^−2^	0		9.66 × 10^−4^
Mgboshimini	*Oreochromis niloticus*	2.36 × 10^−5^	7.31 × 10^−4^		6.60 × 10^−4^	6.01 × 10^−2^	0		2.36 × 10^−4^
Transamadi	*Clarias gariepinus*	5.42 × 10^−4^	0		4.71 × 10^−4^	2.24 × 10^−2^	2.36 × 10^−5^		2.36 × 10^−5^
Transamadi	*Mugil cephalus*	9.43 × 10^−5^	1.65 × 10^−4^		3.77 × 10^−4^	1.18 × 10^−1^	0		1.41 × 10^−4^
Transamadi	*Oreochromis niloticus*	9.43 × 10^−5^	0		5.42 × 10^−4^	1.28 × 10^−1^	0		1.89 × 10^−4^
B. Target Hazard Quotient (THQ)									
**Location**	**Species**	**THQ_Zn**	**THQ_Pb**		**THQ_Fe**	**THQ_Mg**	**THQ_Cr**		**THQ_Cd**
Iwofe	*Clarias gariepinus*	2.36 × 10^−4^	2.95 × 10^−1^		5.72 × 10^−4^	2.28 × 10^−1^	3.93 × 10^−2^		7.07 × 10^−2^
Iwofe	*Mugil cephalus*	5.50 × 10^−4^	0		6.73 × 10^−4^	7.46 × 10^−2^	7.86 × 10^−3^		2.36 × 10^−2^
Iwofe	*Oreochromis niloticus*	7.86 × 10^−5^	1.77 × 10^−1^		3.37 × 10^−4^	5.58 × 10^−1^	6.29 × 10^−2^		3.54 × 10^−1^
Mgboshimini	*Clarias gariepinus*	5.50 × 10^−3^	1.77 × 10^−1^		3.37 × 10^−4^	5.58 × 10^−1^	6.29 × 10^−2^		3.54 × 10^−1^
Mgboshimini	*Mugil cephalus*	1.02 × 10^−3^	8.84 × 10^−1^		1.35 × 10^−4^	2.20 × 10^−1^	0		9.66 × 10^−1^
Mgboshimini	*Oreochromis niloticus*	7.86 × 10^−5^	1.83 × 10^−1^		9.43 × 10^−4^	2.00 × 10^−1^	0		2.36 × 10^−1^
Transamadi	*Clarias gariepinus*	1.81 × 10^−3^	0		6.73 × 10^−4^	7.46 × 10^−2^	7.86 × 10^−3^		2.36 × 10^−2^
Transamadi	*Mugil cephalus*	3.14 × 10^−4^	4.13 × 10^−2^		5.39 × 10^−4^	3.93 × 10^−1^	0		1.41 × 10^−1^
Transamadi	*Oreochromis niloticus*	3.14 × 10^−4^	0		7.74 × 10^−4^	4.28 × 10^−1^	0		1.89 × 10^−1^
C. Hazard Index (HI)									
**Location**		**Species**				**HI**			
Iwofe		*Clarias gariepinus*				0.633			
Iwofe		*Mugil cephalus*				0.107			
Iwofe		*Oreochromis niloticus*				1.151			
Mgboshimini		*Clarias gariepinus*				1.157			
Mgboshimini		*Mugil cephalus*				2.072			
Mgboshimini		*Oreochromis niloticus*				0.620			
Transamadi		*Clarias gariepinus*				0.109			
Transamadi		*Mugil cephalus*				0.576			
Transamadi		*Oreochromis niloticus*				0.618			
D. Cancer Risk (CR)									
**Location**	**Species**	**CR_Pb**		**CR_Cr**		**CR_Cd**		**Total CR**	
Iwofe	*Clarias gariepinus*	1.00 × 10^−5^		5.89 × 10^−5^		2.69 × 10^−5^		9.58 × 10^−5^	
Iwofe	*Mugil cephalus*	0		1.18 × 10^−5^		8.96 × 10^−6^		2.07 × 10^−5^	
Iwofe	*Oreochromis niloticus*	6.01 × 10^−6^		9.43 × 10^−5^		1.34 × 10^−4^		2.35 × 10^−4^	
Mgboshimini	*Clarias gariepinus*	6.01 × 10^−6^		9.43 × 10^−5^		1.34 × 10^−4^		2.35 × 10^−4^	
Mgboshimini	*Mugil cephalus*	3.01 × 10^−5^		0		3.67 × 10^−4^		3.97 × 10^−4^	
Mgboshimini	*Oreochromis niloticus*	6.21 × 10^−6^		0		8.96 × 10^−5^		9.58 × 10^−5^	
Transamadi	*Clarias gariepinus*	0		1.18 × 10^−5^		8.96 × 10^−6^		2.07 × 10^−5^	
Transamadi	*Mugil cephalus*	1.40 × 10^−6^		0		5.37 × 10^−5^		5.51 × 10^−5^	
Transamadi	*Oreochromis niloticus*	0		0		7.17 × 10^−5^		7.17 × 10^−5^	

Note: EDI: estimated daily intake; THQ: target hazard quotient; HI: hazard index (sum of THQs); CR: Carcinogenic Risk. Values < 1 (THQ, HI) indicate no significant non-carcinogenic risk; acceptable CR range: 10^−6^–10.

## Data Availability

The data presented in this study are available on request from the corresponding author. Data are not publicly available due to ongoing analyses.

## Supplementary Materials

The following supporting information can be downloaded at: https://www.mdpi.com/article/10.3390/ijerph23070827/s1, Table S1: Raw metal concentrations (mg/kg) in fish tissues by species and location (n = 3 per group). Table S2: Raw triplicate water quality data (pH, conductivity, turbidity, BOD, salinity) for the three sampling sites (n = 3 per site). Table S3: Complete two-way ANOVA results for all six metals (sheets: ANOVA Cd, ANOVA Cr, ANOVA Fe, ANOVA Mg, ANOVA Pb, ANOVA Zn). Table S4: Pearson correlation matrix for metal pairs across all samples. Table S5: Principal component analysis loadings and eigenvalues for metals. Table S6: Complete health risk indices including estimated daily intake (EDI), target hazard quotient (THQ), hazard index (HI), and carcinogenic risk (CR) per metal. Table S7: Hazard index and total cancer risk summary by species and location. Table S8: Principal component analysis eigenvalues and variance explained for water quality parameters. Table S9: Principal component analysis loadings for water quality parameters. Table S10: Pearson correlation matrix for water quality parameters (pH, conductivity, turbidity, BOD, salinity). File S1: Atomic absorption spectrophotometry instrument parameters and quality control summary. Raw AAS calibration files are not available due to instrument data retention limitations. All calculated datasets are provided in the tables above.
